# Pathways to Diverse Diets—a Retrospective Analysis of a Participatory Nutrition-Sensitive Project in Kenya

**DOI:** 10.1093/cdn/nzab140

**Published:** 2021-11-26

**Authors:** Julia Boedecker, Carl Lachat, Dana Hawwash, Patrick Van Damme, Marisa Nowicki, Céline Termote

**Affiliations:** Food Environment and Consumer Behaviour Lever, The Alliance of Bioversity International and International Center for Tropical Agriculture (CIAT) , Nairobi, Kenya; Department of Food Technology, Safety and Health, Ghent University, Ghent, Belgium; Department of Plants and Crops, Ghent University, Ghent, Belgium; Department of Food Technology, Safety and Health, Ghent University, Ghent, Belgium; Department of Food Technology, Safety and Health, Ghent University, Ghent, Belgium; Department of Plants and Crops, Ghent University, Ghent, Belgium; Food Environment and Consumer Behaviour Lever, The Alliance of Bioversity International and International Center for Tropical Agriculture (CIAT) , Nairobi, Kenya; Food Environment and Consumer Behaviour Lever, The Alliance of Bioversity International and International Center for Tropical Agriculture (CIAT) , Nairobi, Kenya

**Keywords:** nutrition-sensitive agriculture, community-based participatory approach, dietary diversity, agriculture–nutrition pathways, Kenya

## Abstract

**Background:**

There is a current need for better understanding the impact of nutrition-sensitive agriculture interventions. This study is based on a community-based participatory project that diversified diets of women and children by making use of local food biodiversity. This retrospective impact pathway analysis aims at explaining why and how impact was reached.

**Objectives:**

This study aimed to understand how a nutrition-sensitive agriculture project improved people's diets by analyzing the pathways from agriculture to nutrition. It also aimed to test theoretical pathways by comparing the documented pathways with those from a widely used framework from the literature.

**Methods:**

A qualitative study was conducted in 2019 through 10 semistructured focus group discussions with community members engaging in the project and 5 key informant interviews with local authorities that worked with these communities during the project. Summative content analysis was used to identify pathways through which the project affected diets of beneficiaries. The defined pathways were compared with the pathways of the widely used Tackling the Agriculture–Nutrition Disconnect in India (TANDI) framework from the literature.

**Results:**

Out of the agriculture–nutrition pathways that are presented in the literature, 3 were found in the responses: *1*) food from own production; *2*) income from sale of foods produced; and *3*) women's empowerment through access to and control over resources. In addition, 5 other pathways were identified and indicated spillover effects from the intervention to the control participants, increased nutrition knowledge, improved health, savings, and empowerment and harmony in the household.

**Conclusions:**

Pathway analysis in nutrition-sensitive agriculture can provide valuable understanding on how and why dietary improvements have been achieved in an intervention. The approach can hence be instrumental in addressing the current demand within the field on understanding the progress and impact of interventions. Pathway analysis also helps to address knowledge gaps regarding theoretical frameworks, as in the present study, concerning women empowerment pathways.

## Teaser

This study is based on a participatory nutrition-sensitive agriculture project that diversified diets of women and children. A retrospective impact pathway analysis explains how this impact was reached.

## Introduction

Agricultural interventions will have to become nutrition-sensitive to improve food access and attain global nutrition targets (1–3). Nutrition-sensitive agriculture approaches incorporate nutrition objectives, concerns, and considerations to achieve food and nutrition security by drawing on the sectors of agriculture and health. To increase impact, agricultural programs need to be implemented at scale, reaching highly vulnerable populations (4). Evidence from impact evaluations in low**-** and middle-income countries suggests that nutrition-sensitive agriculture interventions can improve both maternal and child nutrition outcomes (5–8). That said, there is a current need to conduct further research beyond impact evaluations. Further studies are needed to understand how and why nutrition-sensitive agriculture programs achieve their outcomes, because a more nuanced understanding of these mechanisms can inform future program design (9–13).

To better understand the impact of nutrition-sensitive agriculture interventions, multiple outcomes, in addition to nutritional outcomes, must be evaluated (1, 10). For example, impact assessments tend to overlook social outcomes that mediate nutrition outcomes, such as women's knowledge, education, social status, and control of resources (14).

Theoretical frameworks have previously been used to better understand the pathways by which agriculture and nutrition are linked. The characterization of these pathways, as well as the mediating role of women's empowerment in these linkages, have been instrumental in stimulating the development of new initiatives that leverage agriculture to improve nutrition (5). In the past 10 y, several conceptual frameworks have been developed that highlight the dynamic and multifaceted linkages between agriculture, health, and nutrition (15–17). One framework developed for the Tackling the Agriculture–Nutrition Disconnect in India (TANDI) project has been used to conceptualize pathways through which the agriculture sector may affect nutrition outcomes (18–20). This framework has been helpful in moving the debate on agriculture's contribution to nutrition beyond the implicit assumption that increases in the output of nutritious foods would be sufficient to improve nutrition; the framework maps 6 pathways that unpack the diverse processes mediating such links between nutrition and agriculture.

It is, however, unclear how well this theoretical framework captures the reality of how an agriculture intervention might actually affect people's nutrition (21). According to a review by Sharma et al. (22), there is limited evidence on the pathways depicting the effect on each temporal stage from nutrition-sensitive agriculture interventions to nutrition outcomes. Several studies in the literature report a knowledge gap regarding the exact functioning of women empowerment pathways in agriculture–nutrition frameworks (5, 22, 23). Moreover, there is a knowledge gap regarding the effect of income gained from agricultural work on nutrition outcomes (22). Contextual understanding is particularly important for programs that aim to simultaneously improve women's status, agricultural production, and nutritional well-being (24).

The present study documents the pathways by which a nutrition-sensitive agriculture project in Kenya improved diets. Since 2015, this project has aimed to diversify diets of women and young children by making use of the local food biodiversity (more information to project in **Box 1**). An impact assessment of the first 2 y indicated that dietary diversity of women and young children had increased, and that the share of children consuming legumes/nuts, flesh foods, and dairy significantly increased (25). That said, more research was needed to fully understand how the project activities led to the improved dietary outcomes.

This study aimed to understand why and how the intervention resulted in improved nutrition outcomes of women and young children. In addition, this study tests theoretical pathways by comparing our documented pathways with the pathways from the TANDI framework. Lastly, this study investigates if the intervention led to possible secondary effects beyond nutrition outcomes, such as factors that increased overall well-being.

The documentation of pathways between interventions and outcomes is valuable because it can inform our understanding of how and why an impact was achieved. Understanding pathways better in their complexity can help maximize the role agriculture can play in achieving long-term nutrition outcomes (22). With the building of understanding of agriculture-to-nutrition pathways in real-world conditions, this research is situated within the field of implementation science. The growing recognition of the critical importance of addressing the “implementation gap” has stimulated interest in developing and applying implementation science in nutrition (26, 27).

BOX 1: In 2015, 36 men and women were selected in each of the 5 intervention sublocations to participate in a series of workshops. One-third of workshop participation was reserved for women with a young child, the second third for male farmers, and the last third for community members whose decision-making role can affect childcare and nutrition decisions (village elders, spiritual leaders, teachers, etc.). The workshops were designed to encourage and support communities in autonomously identifying and planning agricultural activities to improve nutrition, as well as raising awareness on nutrition. At the workshops, all groups identified poultry raising and kitchen gardening (particularly traditional leafy vegetables and legumes) to support dietary diversification. They also expressed strong interest in receiving nutrition education. Through group work, discussions, and presentations, they developed community action plans specifying how the identified activities would be realized. After the workshop series, almost all workshop participants registered themselves as project members, paying a fee that they themselves determined. During the first year of implementation in 2016, the project members received training in kitchen gardening, poultry keeping, and nutrition education. Workshops and implementation of activities were supported by community health volunteers (CHVs) (25).

## Methods

Linkages between agriculture and nutrition are complex and context-specific (28). Therefore, a qualitative, cross-sectional study using focus group discussions (FGDs) and key informant interviews (KIIs) was used for the present study. The qualitative data help understand the motivations and thought processes of people behind a decision (29). In addition, they allow keeping a broader scientific scope to also investigate unintended effects of an intervention. Previous studies have shown how qualitative research with beneficiaries and key informants (KIs) provides plausible explanations for how nutrition-sensitive agricultural interventions bring about changes in communities (1).

### Study site and population

Vihiga County is located in the Lake Victoria Basin of Western Kenya. It is divided into 4 administrative subcounties and further into 9 divisions, 37 locations, and 129 sublocations. Vihiga County mainly lies in the upper midland agro-ecological zone (30). The county's economy is predominantly agricultural, with ∼85% of the population earning their livelihood mainly from agricultural activities. Of the 65% of the population estimated to be living below the absolute poverty line, subsistence farmers account for 90% (31). According to the 2019 population and housing census, Vihiga County has a population density of 1047 persons/km^2^. The dominant ethnic group in the county are the Luhya (32).

### Participants

In each of the 5 sublocations, 2 FGDs were conducted: 1 with the male participants and 1 with the female participants of that sublocation. The CHVs who have supported the project from 2015 developed lists of all male and female project participants per sublocation. Project participants represent the community members who have participated in the introductory workshops (2015), as well as in the implementation of the agricultural activities (2016). From each list, 8 participants were randomly selected for each of the 2 FGDs in that sublocation. Because some of the selected participants did not show up, a final sample of 67 participants in 10 FGDs was obtained.

The FGDs were complemented by KIIs with government and nongovernmental organization employees that have been involved in the project since 2015. The interviewees were affiliated with the local Ministry of Health (2 KIs), the local Ministry of Agriculture (1 KI), the Western Region Agriculture Technology Evaluation (WeRATE) (1 KI), and the Sustainable Organic Farming and Development Initiatives (SOFDI) (1 KI). Individuals with a variety of perspectives on project activities were selected to assess different aspects of the topic, to increase comprehensiveness of the results, and to help interpret the FGD statements. In addition, the 5 chosen individuals represent everyone working on this project in 2015 and 2016. Because the local Ministry of Health was particularly strongly involved, it was decided to choose 2 representatives from this entity.

### Data collection

The FGDs and KIIs were conducted in July 2019. After drafting the FGD interview guide in English, it was translated into the local language (Luhya). Each FGD was conducted by our research team, which included 1 facilitator (female) and 1 note taker (male) who spoke the local language, and 1 Bioversity researcher (female; first author). The FGDs were not audio-recorded because we wanted to encourage participants to feel at ease giving direct and honest feedback. The note taker took handwritten notes. Every evening, the research team reviewed and discussed the notes of the 2 FGDs that were done that day to clarify any ambiguities.

The study was introduced to the FGD participants as an intention to capture changes in their community. In all FGDs, the same 2 very general questions were asked: “Have there been any changes in your community in the past four years?” and, if so, “Which kind of changes?” These general questions were supposed to reduce the subject bias, by not guiding the respondents toward any project activities.

If they mentioned changes related to nutrition and agriculture, the moderator probed for details. If these topics were not mentioned, the moderator asked whether any changes in the field of nutrition and agriculture had occurred. For the FGDs with women, participants were asked about the agricultural and nutritional changes specifically affecting women, whereas male FGD participants were asked about the agricultural and nutritional changes specifically affecting the men. The research team took note of nonverbal communication, e.g., the dynamics among the participants, as well as the level of agreement or disagreement.

The first FGD (with women) was used as a pilot. Because the interview guide did not have to be altered based on the findings of the pilot, the responses from it were included in the analysis (33). Each FGD took ∼2 h.

The Bioversity researcher conducted the KIIs and took handwritten notes. Because all KI interviewees were fluent in English, the interviews were conducted in English. All interviewees were asked the same questions that were asked during the FGDs; they were asked if they saw any changes in the 5 subcommunities. The questions were kept broad so they could discuss male participants, female participants, or both. KIIs took ∼1 h.

### Data analysis

This study aimed to understand why and how the intervention improved the nutrition of women and young children. In addition, this study aimed to test theoretical pathways by comparing our documented pathways with the pathways from the TANDI framework. Moreover, we investigated if our intervention led to possible secondary effects beyond nutrition outcomes, such as factors that increased overall well-being.

The same approach was used to analyze the data from FGDs and KIIs. For the purposes of the study, a “pathway” is defined as follows:

a chain of nodes describing the exact process that possibly lies in between an intervention component (kitchen gardening, poultry keeping, nutrition education) and expected outcome (increased dietary diversity among household members of the intervention sublocations). Nodes represent the basic units of the pathway structure with the purpose to illustrate the main stations within a pathway. A connection between 2 nodes is considered a ‘link.’

Transcripts were analyzed by 2 researchers: a Bioversity researcher and a PhD researcher from Ghent University. Both researchers independently looked at the FGD and KII transcripts. They defined all pathways, and relevant nodes within pathways, leading from the 3 main project activities (kitchen gardening, poultry keeping, and nutrition education) to the increased dietary diversity among the household members participating in the project activities. They also investigated unintended secondary effects of the intervention, which have enhanced the beneficiaries’ overall well-being beyond their nutrition. After analyzing the data from each FGD and KII, the researchers compared their results and reached a consensus. During this analysis process the researchers developed a pathway framework including all nodes they found and agreed upon.

A large number of nodes were identified, leading researchers to create a “summative content analysis” to structure and filter the results. All agreed-on links, pathways, and secondary effects were listed in a table. The researchers went through all FGDs and KIIs again and counted how often each link, pathway, and secondary effect was mentioned. It was also noted whether the statements came from a female or male participant, and from an FDG or KII. Only the contents mentioned at least twice in all FGDs and KIIs were considered for the results. The presented strength of pathways and links is based on how frequently they were mentioned in FGDs and KIIs.

Krueger and Casey (34) provide 5 established criteria that suggest the following headings as a framework for interpreting qualitative data: frequency; specificity; emotions; extensiveness; big picture.

Looking at the transcripts, the frequency with which certain contents are mentioned is striking. Therefore, we measured the weight of a link, pathway, or secondary effect according to the number of mentions. Determining the frequency with which certain objects (or persons, institutions, or concepts) are mentioned is a common strategy for interpreting qualitative data (34–36).

When statements needed clarification or verification, moderators and KIs were consulted via phone. To assess whether the intervention showed any additional links and pathways compared with the ones in the conceptual frameworks from the literature, we compared the simplified framework with a widely used framework developed for the TANDI project (19, 20). This framework shows 6 main pathways from agriculture to nutrition: *1*) food access from own production; *2*) income from the sale of commodities produced; *3*) food prices from changes in supply and demand; *4*) women's social status and empowerment through increased access to and control over resources; *5*) women's time through participation in agriculture; and *6*) women's health and nutrition through engagement in agriculture. Ruel et al. (5) refer to the same 6 pathways (13) in their comprehensive review on nutrition-sensitive agriculture. We adapted the TANDI framework according to our findings, meaning that pathways not applying to our situation were deleted from their framework and additional links and pathways we had found were added.

### Ethics

Ethical clearance was obtained from the Institutional Ethics Review Committee of the accredited University of Masinde Muliro in March 2019. Written informed consent was obtained from all respondents. Survey objectives were explained to village chiefs in order to obtain their permission to conduct the survey in their respective locations. To ensure anonymity, each participant was given a unique number and no personal identifiers were stored.

## Results

This results section is divided into 3 main sections: Respondents’ characteristics; Pathways to diverse diets; and Unintended secondary effects of the intervention. The descriptions of pathways and secondary effects in the latter 2 sections are supported by quotes which were carefully selected for best illustrating the researchers’ conceptual interpretation of the data.

### Respondents’ characteristics

**Table 1** presents respondents’ characteristics per FGD. A mean of 6.7 people participated in each FGD. Women (61%) accounted for a higher percentage in the FGD participants than men (39%). This difference can mainly be explained by the uneven participation in Wanondi, where 12 women participated in the female FGD and only 1 man participated in the male FGD. We decided to interview him on his own.

**TABLE 1:** Sociodemographic characteristics per focus group discussion sample

Sublocation	Gender	*n*	Age, mean ± SD	Married, *n* (%)	Completion of primary and secondary education, *n* (%)
Mambai	Male	7	61.6 ± 7.3	5 (71.4)	7 (100)
	Female	7	35.4 ± 8.9	6 (85.7)	6 (85.7)
Essunza	Male	6	51.3 ± 5.6	6 (100)	6 (100)
	Female	7	45.7 ± 4.8	7 (100)	5 (71.4)
Wanondi	Male	1	49	1 (100)	0 (0)
	Female	12	43.8 ± 15.1	12 (100)	0 (0)
Itumbu	Male	6	32.0 ± 8.3	5 (83.3)	6 (100)
	Female	7	41.3 ± 11.3	4 (57.1)	2 (28.6)
Masana	Male	6	58.2 ± 13.3	6 (100)	3 (50.0)
	Female	8	47.3 ± 9.2	7 (87.5)	3 (37.5)

### Pathways to diverse diets

**FIGURE 1:**
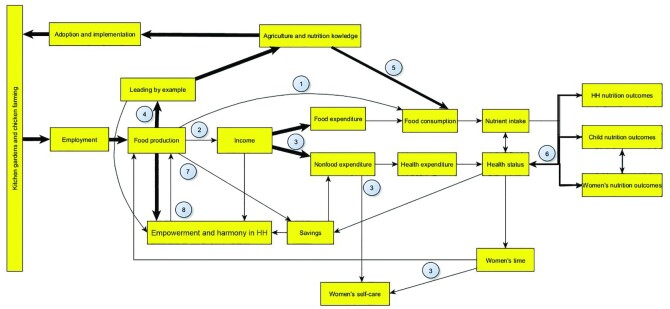
Pathways leading from community-based agricultural activities to improved nutrition outcomes. This figure is the result of the FGDs and KIIs conducted. The numbers indicate the pathways that were found: 1—Food access from own production; 2—Income from the sale of commodities produced; 3—Women's empowerment through increased access to and control over resources; 4—Spillover effect; 5—Nutrition knowledge; 6—Improved health; 7—Savings; 8—Empowerment and harmony in the household. The arrows represent the links within the pathways and differ in width based on their strength, which was measured by the number of times the links were mentioned in the FGDs and KIIs. Very wide links were mentioned 10–21 times, wide links 5–10 times, and thin links 2–4 times. FGD, focus group discussion; HH, household; KII, key informant interview.

This section describes the identified pathways that were derived from our participatory project to improved diets. **Figure 1** presents the framework. The numbers represent the pathways that were found: *1*) Food access from own production; *2*) Income from the sale of commodities produced; *3*) Women's empowerment through increased access to and control over resources; *4*) Spillover effect; *5*) Nutrition knowledge; *6*) Improved health; *7*) Savings; *8*) Empowerment and harmony in the household. The arrows represent the links within the pathways and differ in width based on their strength, which was measured by the number of times the links were mentioned in the FGDs and KIIs. Very wide links were mentioned 10–21 times, wide links 5–10 times, and thin links 2–4 times.

Within the framework, the starting point is represented by the box “Agriculture and nutrition knowledge.” As previously mentioned, project participants received training in kitchen gardening and poultry keeping, as well as nutrition education during the first year of implementation. Their agriculture and nutrition knowledge thus increased, which led to the adoption and implementation of the chosen activities. The livelihoods resulting from these income-generating activities led to increased employment. Employment mostly refers to female employment, because these agricultural activities were culturally viewed as “women's responsibilities.” After starting implementation, it took ∼3 mo to produce food. “Food production” is tied to several pathways and links that led to improved nutrition outcomes.

The FGDs and KIIs indicated unintended secondary effects that had improved the community members’ well-being beyond nutrition. The most prominent of these secondary effects were related to empowerment and improved harmony in the household. Because both empowerment and harmony in the household were directly linked with other nodes, these secondary effects were included in our framework.

#### Pathways commonly known in the literature

##### Pathway 1: Food access from own production

Regarding Pathway 1, Project members and their families directly consumed the farm products from the activities of kitchen gardening and poultry keeping. One woman expressed: “Through the trainings, I know how to keep chicken at home. My children now have eggs to eat and sometimes I can slaughter chicken.”

##### Pathway 2: Income from the sale of commodities produced

Pathway 2 was the most frequently mentioned. As depicted in pathway 2, participants also earned an income from selling the food they produced. A man stated: “I sold cowpeas and mrenda (jute mallow/*Corchorus olitorius*) and got kshs 5000 [∼$50 USD] in one-month sales. I used the money to pay school fees and buy maize flour and sugar.” A male KI mentioned: “Earnings from the vegetable selling is spent on health care for women, which increases women's health.”

##### Pathway 3: Women's empowerment through access to and control over resources

Pathway 3 can be found in several links in our framework. First, women decided on how to spend the income they earned from the agricultural activities. They reported that they spent it on food items (e.g., meat, sugar, maize flour, fish) and nonfood items (e.g., school fees, books, medicine, household utensils). Women also mentioned purchasing some self-care items, such as cosmetic products and clothes. One woman from the FGDs said: “We were taught how to kitchen garden to make some money while at home. I sell the vegetables and buy meat.” Another woman mentioned: “Women sell farm products to buy clothing and can afford to make their hair and afford body oil.” In addition, women decided how to spend the extra time gained from their improved health status (explained below in “Pathway 6”). This newfound time was often spent on self-care or farm work. A female KI noted: “Women have more time to take care of themselves as nutritional status of children improved (don't need to bring them to facilities so much), this time they use for other work, some ventured into farming, most started own kitchen gardens and small animals.”

#### Additional pathways found compared with the ones from the literature

##### Pathway 4: Spillover effect

Pathway 4, an additional frequently mentioned pathway, refers to a spillover effect regarding agricultural activities. Project participants frequently shared their knowledge on kitchen gardening, poultry, and healthy diets with their neighbors, friends, and other community members. Non–project participants often initiated this process, as they approached the project participants when they saw these individuals successfully producing vegetables. Project participants, in turn, were eager to share the newly obtained agricultural and nutritional knowledge. In exchange for their knowledge and guidance, participants were often given seeds from non–project participants for new kitchen gardens. Sometimes this knowledge sharing was initiated by the project participants. For example, project participants taught neighbors how to grow their own food as a protective measure to prevent them from stealing. A woman said: “We teach our neighbors so that they don't steal from us. Currently, there is a lot of vegetables in the community.” In other instances, it was done as a strategic business decision. A male KI explained that if farmers sold vegetables together, they were more likely to be able to sell at bigger markets and generate additional income. Each of these points could explain the participants’ motivation to share their knowledge.

##### Pathway 5: Nutrition knowledge

The nutrition knowledge from the trainings provided encouraged many project participants to consume a more diverse diet and to prepare a more diverse diet for their children. Project participants frequently reported that fruits were now eaten more often, because participants had learned that fruits are a valuable addition to their diets. A man mentioned, “Like avocado. We used to see it and think it has no value, but after the training, we eat them and give to children.”

##### Pathway 6: Improved health

A moderately strong pathway goes from the improved nutrition outcomes to improved reported health statuses. The FGDs and KIIs frequently mentioned that improved diets (especially increased consumption of vegetables) led to improved perceived nutrition outcomes. This again led to an improved perceived health status, and to fewer hospital visits from all household members (especially men and children). A woman from an FGD expressed: “With the TLVs [traditional leafy vegetables], we do not get diseases most of the time. We are healthy and diseases have reduced.”

Improved health status saved time, especially for women who usually take their children to hospital. Women used the time previously used to care for sick family members to take care of themselves. In 1 KI women said they were “taking care of themselves,” which usually meant doing their hair and taking care of their homes. A female KI mentioned: “Women's nutritional status improved as they have time to take care of themselves due to less hospital visits.” Women also chose to spend some of this saved time farming (food production). Fewer hospital visits saved money as families could reduce treatment and transportation costs. Because men would have usually paid for these expenses, they now saved money. A man said: “It has saved me money, I used to visit the hospital often. The incidence of diseases has also gone down in my household after eating these vegetables.” Men partially spent this saved money on nonfood items (e.g., school fees). A female KI stated: “As malnutrition decreased, the costs of treating malnutrition decreased as well in terms of money and time for treatment and transport to the hospitals. Now money is spent on school fees, improving home development and on other farming activities. The saved time is spent for farming.”

##### Pathway 7: Savings

As vegetables were increasingly produced by participants’ own households, expenditures for vegetables reduced. This led to increased savings for men and women, which were used for nonfood expenditures. A woman stated: “We no longer buy vegetables, we save money and use it to pay school fees, make group contributions and make our hair.” A man mentioned: “Now women have been empowered, they know how to plant, some of the vegetables they used to buy they can now pluck from their farms. As a husband, it is a big boost to me. I can save some money because my wife is now independent.”

##### Pathway 8: Empowerment and harmony in the household

Men appreciated the additional vegetables, income, and increased savings that were generated by the kitchen gardening and poultry-keeping activities. This resulted in men increasingly supporting women in these agricultural activities. The new collaboration between men and women in agricultural tasks and income generation also increased harmony in the household. A male KI noted: “Men are sure now that women can generate money and that they can produce seeds and sell. This strengthens men–women relationships, the understanding is more: helping each other.” A woman said: “Our husbands and in-laws are happy; I advise neighbors in types of vegetables to eat to reduce diseases; husbands are happy and support us in our work.” A female KI explained: “Agriculture was a women's job, now we can see partnering between men and women. It has been the cultural belief that kitchen gardening should be done by women. Now more men are involved in it. This is important for farm production as men and women partner together.” A man indicated: “Men now know the importance of supporting women when it comes to vegetable farming.” This collaboration between husband and wife positively affected food production.

### Unintended secondary effects of the intervention

#### Empowerment

The following definition of empowerment was used:

Empowerment is a multidimensional social process that helps people gain control over their own lives. This is a process that fosters power (that is, the capacity to implement) in people, for use in their own lives, their communities and their society, by being able to act on issues that they define as important. (37)

Empowerment increased for both men and women during the course of this project. Owing to the participatory nature of the project, project participants decided how to improve nutrition in their community, gained the respective skills needed to accomplish these goals, and then implemented their chosen activities. In this way, project participants gained more power over their lives, and were able to act on important issues in their communities.

Both male and female FGD participants emphasized that they had come to spend their time more usefully. A man said: “I am now spending my time usefully, my family and I used to loiter a lot.” Another man stated: “The little land that was lying fallow is now in use. I have paved roads in Mombasa but now I have created my own employment. I can pay school fees, medication, and clothing.” A woman noted: “Some women were too idle, now they are busy on the farm.” This can be a sign of participants taking control over how they spend their time and putting it toward activities that they deem important.

Women had increased capacity to provide (more) food to their household, through either farm production or food expenditure (bought with the income generated from their kitchen gardening activities). A woman mentioned, “Some women used to wait for their husbands to bring food but now they can provide food on their own through their farms.” Another woman indicated, “As a wife, I feel I am strong and happy. I no longer wait for my husband to bring everything to the household.” This is an example of women's ability to be more independent and self-sufficient, giving them more control over their own lives.

Female project participants, in particular, gained recognition in their communities for their successful kitchen gardens and poultry units. This acknowledgment came with increased social status and respect, which in turn increased their ability to act on important issues within their communities. One woman recognized, “We have been recognized in the community as farmers.” Another woman said, “Some community members have seen benefits members within the Bioversity team have got and are willing to learn some more. They stated, ‘If you want chickens, go to Bioversity members, you will get chickens in their homes.’”

#### Harmony in the household

Male and female interviewees, from FGDs and KIIs, reported that the project increased happiness within the household, increased husband–wife collaboration, and reduced intrahousehold conflict. Before the project, a major point of conflict in the household was women's need to ask their husbands for money for food expenditures. Now that women had an independent source of income through farming, men had more money left to spend on nonfood items (e.g., school fees). One woman expressed: “There is reduced poverty; we sell vegetables to buy other foods. We are no longer asking for money from our husbands every time and this has reduced conflicts among couples.” A man indicated: “Before knowing how to plant vegetables, women were disturbing us, asking for money to buy vegetables. They no longer ask for money, I come home to get the food ready.” Another man stated, “Conflicts have reduced since we are not being asked for vegetables and food.”

As aforementioned, female project participants increasingly received support on the farm from their husbands. This collaboration between husband and wife increased harmony within the households and the social status of women within the household. They were viewed as partners in income generation, rather than dependent of the husbands.

Female participants spent much more time at home, owing to the nature of the work (growing/selling vegetables at home) and a reduced need to go to the market (as they grew more of their own food). As a result, the household was viewed as more secure. Moreover, women were usually home to prepare dinner at an earlier time, which was appreciated by their husbands. One man from the FGD stated, “There is improved security at home because women are always at home so no one can dare come and steal. My wife now cooks at the right time and when I come from work, I always find her at home. I no longer have to worry about vegetables.”

## Discussion

The present study explored the pathways from a nutrition-sensitive agriculture intervention to improved diets of women and young children. It also tested theoretical agriculture-to-nutrition pathways by comparing our documented pathways with the pathways from the widely used TANDI framework. Moreover, it was investigated if our intervention led to possible secondary effects beyond nutrition outcomes, such as factors that increased overall well-being.

From the pathways of the TANDI framework, 3 were observed as pathways in the present intervention: *1*) Food access from own production; *2*) Income from the sale of commodities produced; and *3*) Women's empowerment through increased access to and control over resources. Our framework identifies 5 complementary pathways in addition to the pathways from the literature, which we named as follows: *4*) Spillover effect; *5*) Nutrition knowledge; *6*) Improved health; *7*) Savings; and *8*) Empowerment and harmony in the household. The most obvious connections between the project and the improved nutrition outcomes among women and children are represented by pathways 1 (Food access from own production), 2 (Income from the sale of commodities produced), and 5 (Nutrition knowledge). The implemented participatory nutrition-sensitive agricultural project led to secondary effects that contributed to well-being beyond improved nutrition, including aspects of increased empowerment and increased harmony in the household.

Not only did project participants benefit, but so did those around them. A previous impact assessment (25) reported increased dietary diversity among female non–project participants in these sublocations. The present qualitative data imply that their improved diets can be related to dissemination regarding nutritional and agricultural knowledge. Project participants were eager to share their knowledge acquired through the project. The participants talked at length about the family, friends, and neighbors that they shared their information with. Although some information sharing was anticipated, the extent of knowledge dissemination among community members was surprising in the present analysis. Moreover, project participants were motivated to share information for several reasons, including concern for community members, increased social status, theft-prevention, seed sharing, creating business partners, etc. It is worth noting that in this community neighbors seemed to know each other well. They had already established a sense of community and a level of cohesion. Results could be different in areas with high levels of community conflict, large wealth gaps, tribalism, an increased value of independence, etc.

The extent to which nutrition education contributed to improved nutrition outcomes was also addressed in the present findings. According to Ruel et al. (5), agriculture and nutrition education need to be linked to address the underlying determinants of maternal and child undernutrition. Our results support this theory, by showing that nutrition education played an important role in increasing consumption of nutritious foods.

The unintended secondary effects that were noted (empowerment and harmony in the household) are linked to other nodes within our framework. They thus contribute to the final outcome of improved diets. This confirms that social outcomes can mediate nutrition outcomes (14), as stated in the Introduction.

Looking at other studies on nutrition-sensitive agriculture interventions, a clear emphasis on women empowerment pathways was noted in the responses. Rao et al. (18) refer to 3 pathways of the TANDI framework when stating that women's work in agriculture may lead to improvements in nutrition (TANDI pathway 4: women's social status and empowerment through increased access to and control over resources) or deterioration (TANDI pathway 5: women's time through participation in agriculture; TANDI pathway 6: women's health and nutrition through engagement in agriculture). Pathways 5 and 6 of the TANDI framework illustrate trade-offs between women working in agriculture, their child-care obligations, and their own nutritional status. Several studies highlight that agricultural programs and interventions demand a large amount of women's time, which in turn reduces their time for childcare duties, health care–seeking behaviors, food preparation, and leisure (24, 38–41).

The present findings show no indication that women from the project had been physically or temporally overburdened and that their increased work in agriculture negatively affected their own or their children's diets. van den Bold et al. (12) reported a similar result. They examined the impact of Helen Keller International's Enhanced Homestead Food Production program in Burkina Faso on women's time use and associations between changes in women's time use and maternal and child health and nutrition outcomes. Despite increasing the time women spent on agriculture, there was no evidence that this contributed to deleterious effects on their own or their children's nutrition.

Rather than feeling overworked, women in the project conveyed that they had time, energy, and motivation to increase their agricultural workload. They also described that they were idle before, but were now spending their time more productively. Many women in this project had the advantage of selling the vegetables from home, which allowed them to act simultaneously as a caregiver to their family. Moreover, the time spent on agricultural activities appears to indirectly save women time in other areas of their lives. At first glance, it might seem like women have less leisure time owing to the agricultural activities. Yet owing to the improved health and nutrition status of their children, they save time taking them to the hospital. In addition, they do not have to go to the market every day to buy vegetables, which also saves them time.

Another possible explanation is that women in the project were spending less time on agricultural activities than in other projects. Farm sizes in Vihiga County are small, which may have limited the amount of time and energy required of the female participants in our project. Moreover, the participatory nature of our project may have mitigated potentially harmful impacts on women. The project participants—most of which were women—chose their own activities. Therefore, it is likely the participants selected activities that seemed manageable. Lastly, it is possible that the increased male support in a traditionally female activity spread the workload across members of the household.

Pathway 4 of the TANDI framework (Women's social status and empowerment through increased access to and control over resources), which is pathway 3 in the framework proposed here, was however quite prominent in our study. According to Kumar et al. (42), women need to have some level of control over their own decisions and be respected within their communities to benefit from any inputs: e.g., income, agriculture, health and nutrition, behavior change communication. This has been the case for the present project. When women gained more control over the decisions involved in agricultural activities (such as the type of crops to cultivate and sell), they became recognized in their community as successful farmers. Moreover, women gained more control over their lives by managing the income gained from agricultural activities. Women also decided on how to spend their additional free time. The nodes related to our pathway 3 (e.g., women's time) are linked to other nodes within our framework and thus contribute to the final outcome. It is, however, not clear whether women's self-care also relates to improved nutrition.

Similarly to our female respondents, women in a qualitative study on nutrition-sensitive agriculture and gender dynamics in Nepal reported increased decision-making power, new knowledge and skills, increased recognition by their family members of their new knowledge and contributions, and self-efficacy as farmers and sellers.

Pathway 3 of the TANDI framework (food prices from changes in supply and demand) did not emerge from the responses. According to a review of impact pathways to nutrition outcomes in nutrition-sensitive agriculture (22), none of the studies reported on this pathway, perhaps because food price has traditionally been considered at the policy rather than intervention level.

This study also has some limitations. A major limitation is that behavior along the pathways was self-reported and not observed. Assessing the behaviors would have required extensive probing during the FGDs and KIIs, beyond what was already being done. These data were not collected because we did not want the interviews to be a time burden for the participants. This limitation was mitigated by having separate FDGs for men and women, as well as KIIs. The separation of these groups was valuable because we had multiple actors independently make statements related to behavior, which therefore mutually confirmed each other.

That said, one of the strengths of this study is that general, open-ended questions were used during our interviews. This enabled us to discover unexpected information about behaviors. Moreover, the open-ended questions limited any potential social-desirability bias. Asking specific questions about empowerment or community cohesion would have likely resulted in affirmative answers (whether true or not). By leaving the questions open-ended, these topics were brought up spontaneously by participants.

Assessing pathways from project-related agricultural activities and nutrition education to improved diets increased understanding on how dietary improvements have happened and helped with documenting the internal dynamics of our intervention. The present findings are an encouragement to assess agriculture-to-nutrition pathways before project start. This will help to prevent harm (e.g., not increasing the agricultural workload of possibly already overworked women) along the pathways and to point out potentials and capacities (e.g., women's motivation to invest more time in profitable agricultural activities). Comparing the pathways before and after the project can provide considerable understanding regarding the changes that occurred toward improved diets.

A systematic mapping of agriculture-to-nutrition pathways in experimental projects could contribute to an understanding on how nutrition-sensitive agricultural projects affect nutrition. It could also offer important insights on why some interventions work and others do not. Pandey and Gautam (43) identified the linkage between agriculture and nutrition in India using the UNICEF framework. This shows that pathways emerging from nutrition-sensitive agriculture interventions can be mapped for a country and that the results are highly relevant for maximizing the role agriculture can play in achieving nutrition outcomes. It also indicates that theoretical frameworks can be used as a guide when comparing pathways of different projects and programs. A comparable analysis was done by Wordofa and Sassi for Ethiopia (44).

Theoretical frameworks in nutrition-sensitive agriculture can have several purposes. For the vast majority, including the TANDI framework, the main purpose is exposition. This means that frameworks are used to visualize concepts and linkages to facilitate reader understanding of text descriptions. Frameworks, however, can also provide a summary of empirical evidence about specific linkages or pathways (28). Even though the purpose of the TANDI framework has not been to summarize empirical evidence, but rather to facilitate the understanding of linkages, it is important that all depicted pathways are well understood. Our study results can help with narrowing the knowledge gap regarding the exact functioning of women empowerment pathways.

Moreover, research on gender and nutrition‐sensitive agriculture has been primarily quantitative, with little qualitative work on how gender dynamics facilitate or impede predefined agriculture-to-nutrition pathways. The present findings provide contextual understanding on the important role of women's empowerment in agriculture–nutrition projects.

Pathway analysis in nutrition-sensitive agriculture can provide valuable understanding on how and why dietary improvements have been achieved in an intervention. The approach can hence be instrumental in addressing the current demand within the field on understanding the progress and impact of interventions. Pathway analysis also helps to address knowledge gaps regarding theoretical frameworks, as in the present study, concerning women empowerment pathways.

## Data Availability

The anonymized data underlying this article (transcripts of focus group discussions and key informant interviews) will be shared on request to the corresponding author.

## Notes

Supported by Deutsche Gesellschaft für Internationale Zusammenarbeit GmbH (GIZ) (to JB) and The Alliance of Bioversity International and International Center for Tropical Agriculture (CIAT) (study design, analysis and interpretation of data).

Author disclosures: The authors report no conflicts of interest.

Abbreviations used: CHV, community health volunteer; FGD, focus group discussion; KI, key informant; KII, key informant interview; TANDI, Tackling the Agriculture–Nutrition Disconnect in India.
